# Rationale, design and objectives of ARegPKD, a European ARPKD registry study

**DOI:** 10.1186/s12882-015-0002-z

**Published:** 2015-02-18

**Authors:** Kathrin Ebner, Markus Feldkoetter, Gema Ariceta, Carsten Bergmann, Reinhard Buettner, Anke Doyon, Ali Duzova, Heike Goebel, Dieter Haffner, Barbara Hero, Bernd Hoppe, Thomas Illig, Augustina Jankauskiene, Norman Klopp, Jens König, Mieczyslaw Litwin, Djalila Mekahli, Bruno Ranchin, Anja Sander, Sara Testa, Lutz Thorsten Weber, Dorota Wicher, Ayse Yuzbasioglu, Klaus Zerres, Jörg Dötsch, Franz Schaefer, Max Christoph Liebau, ESCAPE Study Group, GPN Study Group

**Affiliations:** Department of Pediatrics, University Hospital of Cologne, Kerpener Str. 62, 50937 Cologne, Germany; Department of Pediatrics, University Hospital Bonn, Adenauerallee 119, 53113 Bonn, Germany; Department of Pediatric Nephrology, University Hospital Vall d’Hebron, Pg/Vall d’ Hebron 119-129, 08034 Barcelona, Spain; Bioscientia Center for Human Genetics, Konrad-Adenauer-Straße 17, 55218 Ingelheim, Germany; Renal Division, Department of Medicine, University Freiburg Medical Center, Hugstetter Straße 55, 79106 Freiburg, Germany; Institute of Pathology, University Hospital of Cologne, Kerpener Str. 62, 50937 Cologne, Germany; Division of Pediatric Nephrology, University Children’s Hospital Heidelberg, Im Neuenheimer Feld 430, 69120 Heidelberg, Germany; Department of Pediatrics, Division of Pediatric Nephrology, Hacettepe University Faculty of Medicine, Sihhiye, 06100 Ankara, Turkey; Department of Pediatric Kidney, Liver and Metabolic Diseases, Hannover Medical School, Carl-Neuberg-Strasse 1, 30625 Hannover, Germany; Hannover Unified Biobank, Hannover Medical School, Carl-Neuberg-Strasse 1, 30625 Hannover, Germany; Institute for Human Genetics, Hannover Medical School, Carl-Neuberg-Strasse 1, 30625 Hannover, Germany; Vilnius University Hospital, Center for Pediatrics, Santariskiu, 08406 Vilnius, Lithuania; Department of General Pediatrics, University Hospital Münster, Waldeyerstr. 22, 48149 Muenster, Germany; The Children’s Memorial Health Institute, Al. Dzieci Polskich 20, 04-730 Warsaw, Poland; Department of Pediatric Nephrology, University Hospitals Leuven, Herestrtaat 49, 3000 Leuven, Belgium; Service de Néphrologie Pédiatrique, Hospices Civils de Lyon, Université de Lyon, Hôpital Femme Mère Enfant, 69677 Bron, France; Institute of Medical Biometry and Informatics, University of Heidelberg, Im Neuenheimer Feld 305, 69120 Heidelberg, Germany; Pediatric Nephrology Unit, Fondazione IRCCS Ca Granda Ospedale Maggiore Polic, Via della Commenda 9, 20122 Milano, Italy; Department of Medical Biology, Center for Biobanking and Genomics, Hacettepe University, Ankara, Turkey; Institute of Human Genetics, RWTH University Hospital Aachen, Pauwelsstrasse 30, 52074 Aachen, Germany; Center for Molecular Medicine, University Hospital of Cologne, Robert-Koch-Str. 21, 50931 Cologne, Germany; Nephrology Research Laboratory, Department II of Internal Medicine, University Hospital of Cologne, CECAD Building, Joseph-Stelzmann-Str. 26, 50931 Cologne, Germany

**Keywords:** ARPKD, Ciliopathy, PKHD1, Polycystic kidney disease, Congenital hepatic fibrosis

## Abstract

**Background:**

Autosomal recessive polycystic kidney disease (ARPKD) is a rare but frequently severe disorder that is typically characterized by cystic kidneys and congenital hepatic fibrosis but displays pronounced phenotypic heterogeneity. ARPKD is among the most important causes for pediatric end stage renal disease and a leading reason for liver-, kidney- or combined liver kidney transplantation in childhood. The underlying pathophysiology, the mechanisms resulting in the observed clinical heterogeneity and the long-term clinical evolution of patients remain poorly understood. Current treatment approaches continue to be largely symptomatic and opinion-based even in most-advanced medical centers. While large clinical trials for the frequent and mostly adult onset autosomal dominant polycystic kidney diseases have recently been conducted, therapeutic initiatives for ARPKD are facing the challenge of small and clinically variable cohorts for which reliable end points are hard to establish.

**Methods/Design:**

ARegPKD is an international, mostly European, observational study to deeply phenotype ARPKD patients in a pro- and retrospective fashion. This registry study is conducted with the support of the German Society for Pediatric Nephrology (GPN) and the European Study Consortium for Chronic Kidney Disorders Affecting Pediatric Patients (ESCAPE Network). ARegPKD clinically characterizes long-term ARPKD courses by a web-based approach that uses detailed basic data questionnaires in combination with yearly follow-up visits. Clinical data collection is accompanied by associated biobanking and reference histology, thus setting roots for future translational research.

**Discussion:**

The novel registry study ARegPKD aims to characterize miscellaneous subcohorts and to compare the applied treatment options in a large cohort of deeply characterized patients. ARegPKD will thus provide evidence base for clinical treatment decisions and contribute to the pathophysiological understanding of this severe inherited disorder.

## Background

Autosomal recessive polycystic kidney disease (ARPKD) is a rare, but severe form of polycystic kidney disease with unexplained phenotypic variability and a considerable impact on affected patients and families as well as attending physicians. The disease is caused by mutations in a single gene, *Polycystic Kidney and Hepatic Disease 1* (*PKHD1*) encoding a huge transmembrane protein of poorly understood function called fibrocystin (4074 amino acids) [1]. Fibrocystin localizes to primary cilia of cells, classifying ARPKD as ciliopathy [2].

Within the last years basic science approaches have brought tremendous progress in the understanding of the genetic basis and pathomechanisms of ciliopathies [2-5]. As a consequence first clinical trials on potential treatment approaches for the common adult-onset autosomal dominant polycystic kidney disease (ADPKD) have been conducted and have aroused much scientific attention [6-12]. Still, these trials have not yet established a clear-cut and safe general treatment therapy for ADPKD.

With an estimated incidence of 1 in 20.000 live births ARPKD is much rarer than ADPKD and lacks similar targeted therapeutic approaches. Furthermore, initiatives for randomized clinical trials on ARPKD are facing the difficulty of clinically highly variable cohorts for which reliable clinical study end points are hard to establish. The underlying molecular mechanisms of the observed clinical heterogeneity are currently poorly understood. Genotype-phenotype correlations are confined to an interrelation of two truncating mutations with a mostly severe phenotype with peri- or neonatal demise and the observation that most patients surviving the neonatal period carry at least one missense mutation as milder manifestation [13,14]. Mutations in modifier genes may be involved as it has been described in other ciliopathies [2]. Finally, *PKHD1* is subject to a complex splicing pattern, adding another level of complexity to mechanisms controlling fibrocystin expression [15,16].

Clinically, the phenotypic spectrum of ARPKD ranges from severely affected newborns with a persisting high mortality of up to 30% often related to respiratory failure in pulmonary hypoplasia [17] to mild affected adults with predominant hepatic fibrosis.

Renal involvement in ARPKD is characterized by bilateral massive enlargement of the kidneys and microcystic kidney disease as a consequence of fusiform tubular dilatations usually deriving from the collecting duct. Kidney function deteriorates over time and kidney transplantation is required in up to 50% of patients within the first two decades of life [14].

Beneath renal involvement ARPKD obligatory encompasses congenital hepatic fibrosis (CHF) due to ductal plate malformation, with or without intrahepatic bile duct dilatation. Portal hypertension, potentially resulting in variceal bleeding, and cholangitis are leading clinical manifestations of liver involvement. Additional clinical manifestations include pronounced arterial hypertension, secondary pulmonary hypertension, and hyponatremia. For a detailed review of the broad spectrum of clinical manifestations we refer to the recent excellent review articles by Büscher et al. [18], Sweeney and Avner [19] and Hartung and Guay-Woodford [15].

Therapeutic management of the various challenging clinical problems is mainly based on the physician’s clinical experiences and from retrospective data or expert opinions, such as the recently published consensus recommendations on ARPKD treatment [20]. In summary, the management of ARPKD currently remains largely opinion-based.

Regarding the description of clinical courses we can refer to important previous studies by Guay-Woodford [17], Adeva [21] and Bergmann [14] who described American and European cohorts. Still, as previously pointed out, there are multiple remaining questions and despite the mentioned expert opinion recommendations [20], there is an urging need e.g. for evidence-based management guidelines and clinical descriptions of long-term follow-ups. Clinical or biochemical risk markers have not yet been established and current treatment approaches have not been systematically reviewed. Finally, the insights obtained from molecular studies on ciliopathies have opened a new understanding of the underlying pathomechanisms. These insights may sharpen our clinical view for subtle phenotypes and may help to classify defined subgroups of ARPKD patients in the future. As nicely pointed out by the Consortium for Radiologic Imaging Study of PKD (CRISP) in a very recent study, classification into different subgroups in ADPKD may have prognostic implications [22]. This may also apply for ARPKD.

To address these open questions on ARPKD two renowned clinical research consortia in pediatric nephrology, i.e. the German Society for Pediatric Nephrology (GPN) and the European Study Consortium for Chronic Kidney Disorders Affecting Pediatric Patients (ESCAPE Network) recently teamed up to start a joint initiative for a clinical registry study exclusively devoted to ARPKD. This registry study (ARegPKD) is now open for the inclusion of patients. Here, we present the details of ARegPKD for the first time.

## Methods/Design

### Study population

ARegPKD is an international, multicenter, pro- and retrospective, observational study in both pediatric and adult ARPKD patients. Inclusion criteria comprise the diagnosis of ARPKD by histology, molecular assessment or clinical evaluation according to the criteria of Zerres et al [23]. Those criteria are a) typical findings on renal imaging and b) one or more of the following criteria:

- Clinical/laboratory signs of hepatic fibrosis
- Hepatic pathology demonstrating ductal plate abnormality
- Absence of renal enlargement and/or multiple cysts in both parents
- Pathoanatomic diagnosis of ARPKD in an affected sibling
- Family history consistent with autosomal recessive inheritance.

Definite genetic, histological or clinical proof of other cystic kidney disorders represents an exclusion criterion. Patient inclusion is preceded by informed consent and by approval of the registry study by a corresponding local ethics committee, thereby guaranteeing accordance with the ethical standards laid down in the 1964 Declaration of Helsinki.

### Data handling

All data will be pseudonymously entered into a password-restricted web-based database on a safe server of the University of Cologne and secured by use of SSL-connections (www.aregpkd.org). Subject identification will only be possible at the local study site and participating centers are exclusively able to review and modify data of patients from the corresponding site. Transmitted data will be stored in the central database on a central and safe server at Cologne University Computing Facilities.

### Data quality

In addition to automated entry checks using predefined plausibility ranges, mandatory statements regarding diagnosis criteria will minimize the risk for incomplete or erroneous data entries as well as inclusion of falsely diagnosed patients. Data entries will also be reviewed by a pediatric nephrologist at random checks and during regular interims analyses. Queries will be sent to the local investigators in cases of implausibility or doubt. In case of major problems with accomplishing regular local data entries, an associated regional coordinator in charge will provide support. Regional coordinators will be named for a group of centers.

### Statistical analysis

Statistical analysis will mainly focus on descriptive analysis including calculation of relative and absolute frequencies for binary/categorical variables and for continuous variables calculation of mean, standard deviation, median, interquartile range, minimum and maximum. Inferential analyses will be planned based on first results of intermediate analyses and predefined in detail in a statistical analysis plan (SAP). Further longitudinal data regarding outcomes of markers of renal and hepatic function, overall survival and e.g. kidney size will be analyzed using appropriate statistical methods such as mixed modeling. In case of missing data appropriate methods (e.g. multiple imputation) will be used to handle them. Furthermore the aspect of multiple testing will be considered. Potential sources of bias will be examined and considered in the analyses.

### Study organization and design

**Figure 1:**
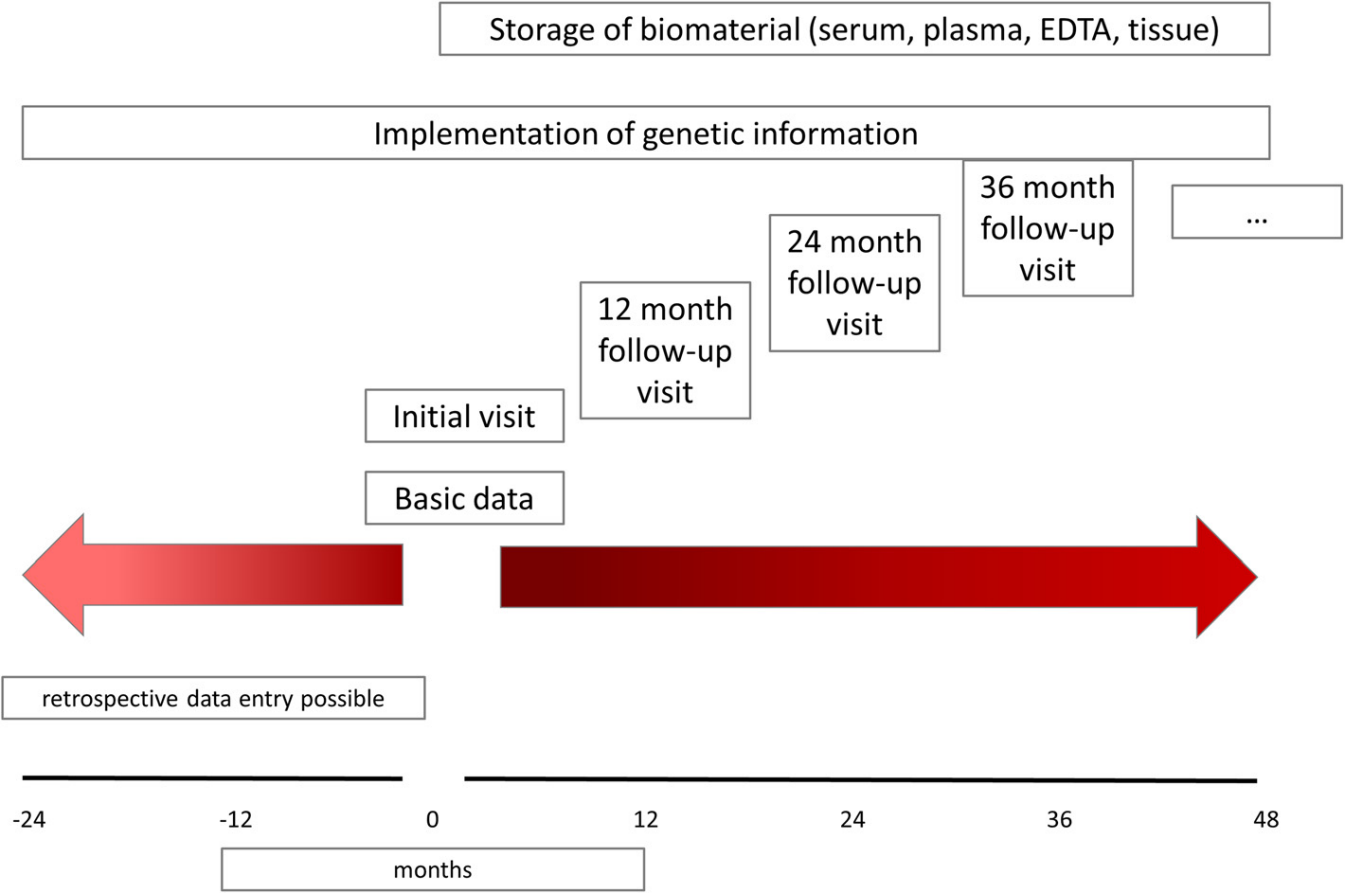
**Study flow chart.**

Data acquisition is partitioned in basic data and follow-up visits (Figure 1).

*Basic data (Table 1)

**Table 1:** **Synopsis of data obtained in ARegPKD**

***Synopsis of data obtained in ARegPKD***	
**Basic data**	
a	Basic information with date of birth, sex, onset of symptoms and initial visit at doctor
b	Family history
c	Pre- and perinatal period/infancy
d	Initial diagnosis
e	Genetics
**Initial visit/yearly follow-up visits**	
a	Patient’s status – baseline evaluation (e.g. body-measurements, participation in studies, availability of biosamples)
b	Renal status including symptoms, radiological findings, biopsy results
c	Extrarenal status regarding liver (symptoms, radiological findings, biopsy results) and other organs (spleen, cardiovascular system, central nervous system, eyes, lungs, bones, blood)
d	Laboratory values
e	Medications with start and end date, dose
f	Therapy including renal replacement therapy, surgical procedures and other procedures
g	Further developments with inclusion of user-defined comments

Within the section of basic data details regarding clinical symptoms at primary manifestation are documented. Available results of genetic testing and family history data regarding cystic kidney disease as well as potential liver affection are included.

*Initial visit/follow-up visits (Table 1)

After completion of basic data the initial visit is created for enrollment of clinical, radiologic, laboratory and other findings of different organ systems (kidney, liver, spleen, cardiovascular system, lung, eye, central nervous system, other organs). A patient’s medication is documented with dose and application duration, surgical procedures are provided with date, indication and possible complications, and histological findings can be entered. Individual aspects not specifically requested in the questionnaire can be added as free text. At least yearly follow-ups with identical questionnaires provide data on the clinical course in a pro- or retrospective manner.

### Biobanking

Blood and tissue samples shall be taken at every visit (both initial and/or follow-up visits). Initial blood samples include e.g. serum, plasma and EDTA blood. Yearly follow-up blood samples (e.g. serum, plasma) shall be collected if possible. Importantly, there will be no additional interventions or blood takings, material for storing will exclusively be obtained within routinely indicated examinations. Samples will be pseudonymously sent to Hannover Unified Biobank (HUB) the central biobank of Hannover Medical School. At HUB the biomaterials will be identified by a unique Lab-ID. HUB is one of the most modern biobanks in Europe. It is characterized by a high degree of automation and state-of-the-art biobank infrastructure according to standard operating procedures with data safety concepts, pseudonymization tools, sample identification via 2-D bar codes and integrated IT systems that will enable high quality and safety standards. Stable biobank structures, continuous temperature monitoring, emergency power supply, and 7/24 sample maintenance guarantee a high safety standard in the short- and long-term storage of biomaterials.

The samples from Turkey will be pseudonymously stored at Hacettepe Biobank at the Biobanking and Genomics Research and Application Center of the Hacettepe University.

Biosamples can be requested for basic research questions by written application to the ARegPKD steering committee and must include a proven positive ethics votum. Appropriation of biosamples will be restricted to research on cystic kidney diseases.

### Reference histology

Reference histology of biopsy samples is offered by the Institute of Pathology of the University Hospital of Cologne. Professor Reinhard Buettner, chair of pathology at the University Hospital of Cologne, is an internationally recognized expert for ARPKD. In addition to the assessment of existing genetic analyses, reference histology evaluation complementing local histological examination can serve as an important factor to maintain highest security and standards concerning the correct diagnosis of ARPKD. Histologic evaluation will focus on the assessment of liver and kidney but will include, if available, samples from other organs. Histological samples will be scanned by a Mirax (Zeiss) slide scanner and uploaded to a central server connected to a web-based virtual histology discussion platform. This will save and store all histological samples in a pseudonymous fashion and make them available for discussion and inspection for all members of ARegPKD.

### Ethics aspects

The study protocol including patient information and consent forms has been reviewed and approved by the Ethics Commission of the Faculty of Medicine of Cologne University. Corresponding local review boards are approached for confirmation of this approval.

Appropriate measures are used to guarantee maximal data confidentiality. All patient-related clinical data is pseudonymized locally. Blood samples are double-coded and neither laboratories nor the central office are able to identify individual patients.

## Discussion

ARPKD is one of the most severe pediatric renal diseases presenting with a substantial and poorly understood phenotypic variability. Still, current management approaches remain opinion-based [20], clinical or biochemical markers for disease progression are missing, and long-term courses have not yet been reviewed in large cohorts.

To promote and elaborate our understanding of ARPKD, the GPN and the ESCAPE Network jointly initiated an international registry study to pool data regarding ARPKD clinical courses as well as different management approaches in European countries. This database is supposed to serve as a basis for translational research. Due to networking of two European consortia ARegPKD is expected to collect one of the largest cohorts of ARPKD patients with longitudinal data acquisition.

A prominent strength of the study will be the coverage of the major proportion of European ARPKD patients treated in highly specialized centers. Patients will be characterized in a pro- and retrospective manner with detailed questionnaires. We aim to get data for a deep phenotypical characterization that may help to identify specific subcohorts of ARPKD patients. Work by various independent groups addressed the question of potential differences in hepatic and renal disease progression in ARPKD [14,17,24]. Our data may also become helpful to give insights into this field.

Precise clinical characterization will be refined by establishing a concomitant ARPKD specific biobank and reference histology. This approach will set basis for translational and clinical research within a phenotypically deeply-defined international cohort of patients.

As a registry study, ARegPKD will have substantial limitations. Potential infirmities could arise from varying data quality with respect to completeness and accuracy of data, especially in case of retrospectively obtained data. Investigator-dependency may be an issue for clinical or radiologic examinations. Registered patients will furthermore show variable extent of genetic testing with multiple techniques; in some patients without genetic testing it may pose a difficulty to uncover phenotypic overlaps (“phenocopies”) with other polycystic kidney disorders. We will in most cases not have access to data on ARPKD-fetuses in case of induced termination of pregnancy. Finally, data may be missed or limited in cases of perinatal or early neonatal mortality contributing to a selection bias that will also be seen due to the character of the participating centers.

Factors influencing disease course like the development of end stage renal disease (ESRD) or hepatic insufficiency have not yet been established. Current clinical management focuses on symptomatic treatment in an opinion-based manner. We are therefore interested in long-term data regarding e.g. the time to ESRD and the effect of sufficient control of arterial hypertension on the deterioration of renal function. The frequently applied RAAS-blockade with angiotensin converting enzyme inhibitors or angiotensin receptor blockers is based on preliminary evidence from animal models [25-27] and from the data on the positive effect of strict blood pressure control on renal failure in children obtained in the ESCAPE study [28]. Still in this study only a minor fraction of patients suffered from ARPKD. The effect of different pharmacological antihypertensive classes on cyst respective kidney size and renal function remains to be elucidated as well as the proof of efficacy of antihypertensive therapy on slowing progression to end-stage-renal-disease in the ARPKD-cohort. Parameters with accelerating or slowing influence on development of renal failure are still to be identified in the ARPKD-cohort.

Additionally, we expect longitudinal data regarding the impact of uni- and bilateral nephrectomy on dialysis regimen and -efficacy, as well as data on the frequency of potentially recovering kidney function within the first year of life. As recently pointed out the currently available level of evidence on the effects of nephrectomy in ARPKD children is very limited [20]. Furthermore, single-center reports regarding outcomes of and indications for simultaneous vs. sequential liver and kidney transplantation in individuals with severe renal and hepatic phenotype [29-31] require analyses in a larger ARPKD cohort as basis for decision-making.

For the common ADPKD recent international clinical trials have studied the effect of different therapeutic approaches on total kidney volume (TKV) and renal function (mTOR inhibitors, vasopressin V2-receptor antagonists, somatostatin analogues, statin [6-10]). In these trials TKV serves as a surrogate parameter for disease progression as data from the CRISP study have shown that increased TKV is associated with worse outcome in ADPKD [32]. Similar attempts for clinical trials in ARPKD are missing. There is limited experimental data on the role of mTOR activation in ARPKD [33-36]. Animal data in PCK rats regarding vasopressin V2-receptor antagonism and its effect on reducing kidney weight and cyst volume is more robust [37,38]. The transfer of the data and studies regarding cyst growth in ADPKD on a cohort of ARPKD patients seems to be obvious and sonographic assessment of kidney length is used as a surrogate of TKV in the ARegPKD questionnaires. Still, this transfer is hampered by the fact, that TKV has not been established as an indicator of renal function deterioration in patients with ARPKD. Furthermore, TKV seems to be no good marker for kidney function in ARPKD patients as the most rapid growth of kidney volume is usually seen in the early disease course while no continuous volume gain is observed during progressive disease with loss of renal function. As TKV cannot be used potential clinical trials will therefore need to establish novel and specific clear-cut primary end points for ARPKD. ARegPKD aims to address this issue by detailed characterization of long term patient courses to set roots for the establishment of clinical trials on targeted treatment approaches.

In summary ARegPKD is a novel web-based international registry study aiming to provide evidence base for clinical treatment decisions and contributing to the understanding of ARPKD.
